# Trazodone-induced parkinsonism in a middle-aged male: A case report

**DOI:** 10.4314/ahs.v24i1.25

**Published:** 2024-03

**Authors:** Hossam Tharwat Ali, Ziad Ashraf Soliman, Firas Aborigiba, Ana Leticia Fornari Caprara, Jamir Pitton Rissardo

**Affiliations:** 1 Qena Faculty of Medicine, South Valley University, Egypt; 2 Faculty of Medicine, University of Tripoli, Libyan Arab Jamahiriya; 3 Federal University of Santa Maria, Medicine Department, Brazil

**Keywords:** Trazodone, triazolopyridine, parkinsonism, movement disorder, drug-induced

## Abstract

**Introduction:**

Trazodone is an antidepressant agent approved for treating major depressive disorders and is also prescribed for insomnia due to its sedative effect. In a few cases, trazodone was associated with parkinsonism. Herein, we describe a case of parkinsonism after a brief exposure to a moderate dose of trazodone.

**Objective:**

To describe a case of a patient with trazodone-induced parkinsonism in which the diagnosis was suspected after the exclusion of other common and serious causes.

**Methods:**

A case report of trazodone-induced parkinsonism.

**Clinical Case:**

A 58-year-old male with sleeping problems was prescribed trazodone 50 mg daily at bedtime. The subject doubled the dosage without medical advice a week later. After 14 days of trazodone treatment, he started to experience difficulty in moving his upper limbs and recurrent falling. Neuroimaging, electrodiagnostic studies, and laboratory exams were unremarkable. Trazodone was discontinued, and the patient fully recovered. Noteworthy, the patient developed a recurrence of the motor symptoms with trazodone-rechallenge.

**Conclusion:**

Our case showed reversibly induced parkinsonism after a short intake of a moderate dose of trazodone which was prescribed for insomnia. The patient had a complete recovery after trazodone withdrawal. Noteworthy, the symptoms recurred upon trazodone-rechallenge.

## Introduction

Trazodone is a triazolopyridine derivative that was first synthesized in Italy. In 1981, trazodone was approved by the United States (US) Food and Drug Administration for managing major depressive disorder. 1 Trazodone is considered an atypical antidepressant that is sometimes prescribed off-label for sleep disturbances, but there is a lack of evidence regarding its safety profile. 2 Parkinsonism is characterized by three cardinal motor manifestations, which are bradykinesia in addition to rigidity and rest tremors. 3 This syndrome can occur as a primary neurodegenerative process, Parkinson's disease, or secondary to other causes such as drugs. In this context, many substances can contribute to parkinsonism, especially those affecting the dopaminergic system either directly or through the serotoninergic pathway. Some case reports hypothesized that trazodone might have an anti-dopaminergic effect. To the authors' knowledge, there are five reports of parkinsonism secondary to trazodone in the literature.^2^,^4^–^7^ Herein, we present a case of a male who developed parkinsonism after a short-duration intake of a moderate dose of trazodone.

## Clinical case

A 58-year-old male came to the emergency department (ED) complaining of sub-acute onset of rigidity in bilateral upper extremities that were associated with falling for the past few days. Medical history included sleeping problems for which a trial of zolpidem 10mg at bedtime was initiated but later interrupted due to dizziness. Then, trazodone 50 mg was prescribed once daily at bedtime. Seven days later, the subject increased the dosage to two tablets of 50 mg at bedtime without medical advice. On day 14 of trazodone treatment, the individual experienced difficulty moving his upper limbs, associated with recurrent falling. Past medical history included hypertension which was controlled with losartan 50 mg. There was no relevant family history of neurological diseases. The patient had no symptoms related to cardiovascular, respiratory, gastrointestinal, or genitourinary systems.

Upon the clinical examination, the subject was fully conscious and oriented to time and place. His vital signs were stable in the ED. Facies was atypical. On neurological examination, there were mild resting tremors, bradykinesia, and rigidity of both upper limbs. En bloc turning and forward flexion of the trunk were observed. Muscle strength was 5/5 strength in all four extremities. Deep tendon reflexes were graded 2+ (brisk response). Sensory and cranial nerve examinations were negative. There were no cerebellar signs. The remaining physical examination was normal.

Laboratory investigations included complete blood count, renal function, liver function, total bilirubin, direct bilirubin, serum electrolytes, erythrocyte sedimentation rate, C-reactive protein, hepatitis serology; HBsAg and HCV, VDRL, HIV-1, and HIV-2 which were all within normal levels. Urinalysis was normal. Cerebrospinal fluid showed 60 mg/dl of glucose (92 mg/dl plasma glucose), 0 white blood cells, 0 red blood cells, and 30 mg/dl of protein. Imaging studies were also obtained. Cranial computed tomography (CT) and brain magnetic resonance imaging (MRI) were done to exclude structural lesions and came back normal. Electroencephalography (EEG) was normal without background epileptic activity.

The differential diagnosis for such cases could be stroke and neurodegenerative diseases which both were excluded after the laboratory tests and imaging studies were all normal. Due to the temporal relationship between the occurrence of symptoms and trazodone intake, trazodone was suspected as the underlying cause.

Finally, the patient was admitted to the hospital to discontinue trazodone under medical supervision. During his admission, a significant improvement in his initial symptoms was observed. On the third day, the patient took a trazodone tablet at night due to insomnia as he was without his sleep medications for two days. Unfortunately, he experienced bradykinesia, tremors, and rigidity in the early morning. Trazodone was withdrawn. The next day, the patient recovered with no residual symptoms and was discharged home. In the follow-up one month and six months later, the patient didn't report any new motor symptoms.

## Discussion

Trazodone has been widely used as an antidepressant drug for decades. It can also help patients with insomnia due to its sedative effect. The exact mechanism of trazodone's sedative effect is not clearly understood. However, trazodone can interact with histaminic (H1) and adrenergic (alpha 1) receptors, which are well-known neurotransmitters related to arousal. Moreover, this phenylpiperazine derivative blocks serotonin pathways, partially explaining its sedating qualities.^8^ Most of the side effects associated with trazodone are mild, except for some cases of priapism, QT interval prolongation, and seizures. 4

Extrapyramidal symptoms (EPS) are among the most common adverse events that patients experience probably due to alteration in the dopaminergic system 9. It was first described in 1952 after chlorpromazine-induced symptoms resembling Parkinson's disease. First-generation antipsychotics such as haloperidol are the most common medications associated with EPS. However, they occur less frequently with atypical antipsychotics, serotonin reuptake inhibitors, and anti-emetics 2,9. A variety of extrapyramidal symptoms has been reported to occur due to trazodone intake including dystonia and parkinsonism which occur acutely and shortly after initiation of trazodone. Furthermore, chronic manifestations such as tardive dyskinesia and tardive akathisia have been also reported 2,10–13. The symptoms of EPS can be debilitating and interfere with daily life and social communication 9.

**Figure 1:**
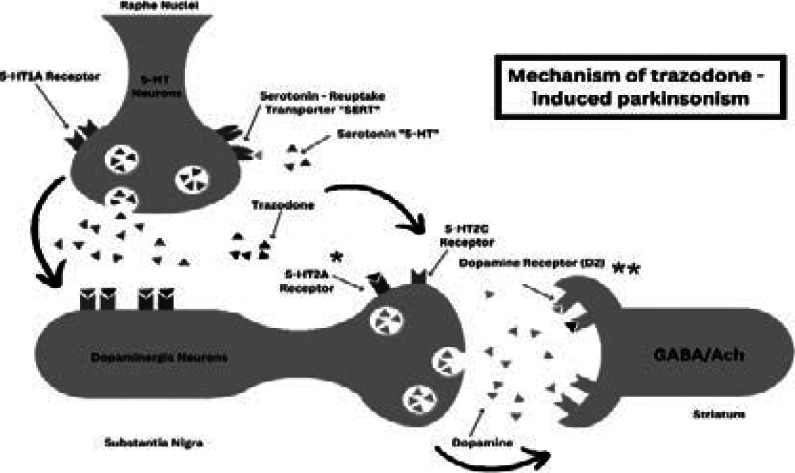
Mechanism of trazodone-induced Parkinsonism. Antagonism of trazodone is more robust at 5-HTA2 receptors than 5-HT2C receptors. Striatal dopamine receptor (D2) blocking activity of trazodone was shown only in rats

A possible explanation for trazodone-induced parkinsonism can be related to dopaminergic pathways (Figure 1). 4 Kapur *et al* suggested that the serotonin system can inhibit dopaminergic neurons at the level of the midbrain and terminal fields in the forebrain. 14 Noteworthy, activation of 5-HT2A receptors may cause enhancement of dopaminergic transmission, while activation of 5-HT2C receptors can inhibit the tonic and evoked dopamine secretion.^15^ Trazodone can antagonize 5-HT2A receptors 15 times more than 5-HT2C. 2 This finding is clinically supported by studies showing increased prolactin levels in depressed patients using trazodone. 16 It is believed that dopamine exerts an inhibitor tone over the prolactin-secreting cells. So, increased prolactin levels mean decreased inhibitory tone of dopaminergic neurons. 17 Such as with other antidepressants, there are case reports of galactorrhea related to trazodone in the literature. 18 Moreover, animal model studies revealed that high doses of trazodone are associated with post-synaptic dopamine (D2) receptor blockage, which was mainly observed in the striatum. 19 Thus, the mechanism of trazodone-induced parkinsonism is probably through the inhibition of both 5-HT2A and D2 receptors which both lead to a decrease in dopaminergic transmission.

So far, trazodone has been found to induce reversible parkinsonism in five reported cases (Table 1). Four of the five cases were males. Four cases were elderly. Trazodone was prescribed for depression in two cases and insomnia in three cases. Motor symptoms gradually developed in the two patients with depression, requiring long-term duration to become apparent. In two other non-depressed cases, symptoms were reported after a shorter duration (2 days a week) despite the moderate trazodone dose (100 mg and 150 mg daily). Both patients were elderly males with comorbidities, e.g., diabetes, hypertension, and restless legs syndrome. The fifth individual reported symptoms after a month despite a low trazodone dosage (50 mg/day). This was probably due to a drug interaction between amiodarone and trazodone. In all cases, patients fully recovered after trazodone discontinuation.

**Table 1:** Reported cases of trazodone-induced parkinsonism

Reference	Sex, age (years)	Indication for trazodone	Dose and frequency of Trazodone	Time from starting of trazodone until first initial symptoms of Parkinsonism	Management of the case	Follow up	Special notes regarding the case
Ali *et al* (The present case)	Male, 58	Insomnia	50 mg/day (for 7 days) + 100 mg/day (for 7 days)	14 days	Admission. Exclusion of stroke and other conditions. Trazodone withdrawal	1 month and 6 months after discharge, complete recovery	Patient was hypertensive and on losartan. Losartan is metabolized by CYP3A4 and so is trazodone.
Sharma *et al.* 2022 (4)	Male, 78	Chronic insomnia	50 mg nightly	A month	Admission for 2 days. Exclusion of stroke and other conditions. Discontinuation of trazodone.	A week after discharge, tremors, and fasciculation resolved.	The patient had comorbidities e.g. cardiac, chronic kidney disease, major depressive disorder, and was on medications such as amiodarone and bupropion. Trazodone is metabolized via CYP3A4 and amiodarone is a CYP3A4 inhibitor.
Sarwar 2018 (2)	Male, 81	Insomnia	150 mg daily	A week	Trazodone taper. Melatonin for sleep difficulty.	Complete resolution of symptoms by the 4^th^ week after trazodone stoppage.	The patient had diet-controlled diabetes mellitus and restless leg syndrome and was on pramipexole and low-dose clonazepam but didn't have depression
Mayor *et al* 2015 (5)	Male, 84	Insomnia	100 mg nightly	2 days	Biperiden intravenously. Trazodone was discontinued.	Complete resolution after biperiden infusion.	The patient had hypertension and was on perindopril, indapamide, and acetylsalicylic acid but didn't have depression
Fukunishi *et al* 2002 (6)	Male, 57	Major depressive disorder	100 mg daily	Months (gradual)	Trazodone was discontinued.	Improvement of symptoms a week after trazodone stoppage.	The patient had end-stage renal disease and was on hemodialysis for more than 8 years.
Albanese *et al* 1988 (7)	Female, 74	Reactive depression	150 mg twice daily	2 months (gradual)	Trazodone discontinuation	Gradual disappearance of symptoms till 14 months after trazodone stoppage, complete recovery.	

**Abbreviations:** CYP3A4; cytochrome P3A4

Our case is unique because it shows the rapid development of parkinsonism in a middle-aged non-depressed male with a moderate dose of trazodone. The previous non-depressed cases were all elderly with/without comorbidities which might have been associated with a lower tolerance to changes in the serotonergic-dopaminergic system.

Our case demonstrates parkinsonism symptoms after a brief exposure to a moderate dose of trazodone in a male adult aged 58 years old. The patient was diagnosed with parkinsonism after excluding other possible structural or metabolic causes. Due to sub-acute onset, a secondary cause of parkinsonism was suspected. The patient was not taking any new drugs other than trazodone at the time of the development of parkinsonism symptoms. Moreover, there was a temporal relationship between trazodone initiation and motor symptoms' appearance. During the follow-up, the patient recovered completely after trazodone withdrawal. Thus, Parkinson's disease or primary parkinsonism was ruled out. In such cases, dopamine transporter (DAT) single-photon emission tomography (SPECT) can differentiate between Parkinson's disease and conditions without presynaptic dopamine deficit, such as drug-induced parkinsonism. 20 Nevertheless, this neuroimaging technique was unavailable at the authors' hospital.

In our case, the patient was on losartan 50 mg twice daily for hypertension. Losartan is metabolized mainly by cytochrome (CYP) 3A4, 2C9, and 2C10 isozymes. 21 Trazodone is metabolized by CYP3A4. 8 We suggest that competitive inhibition of the cytochrome (CYP) 3A4 occurred. This is supported by the fact that the symptoms may have appeared rapidly after the increase of the dose of trazodone from 50 mg (low dose) to 100 mg (moderate dose) daily. Moreover, the symptoms resolved completely shortly after the drug was stopped. Based on the literature, pharmacokinetic interactions with losartan can be CYP3A4-mediated. 22 However, this remains a theoretical explanation as there is no evidence of possible interactions between trazodone and losartan in pharmacological studies. Our study raises awareness of drug interactions and proposes early therapeutic interventions.

Recommendations for patients who are to use trazodone should include strict compliance with the prescribed dose and revising the physician in case any side effects occur. Physicians should avoid prescribing trazodone in high doses and start with the lowest doses. They may try safer alternatives if there are any. Researchers should try to elaborate on the exact mechanism by which trazodone can cause movement events such as parkinsonism. Moreover, they should determine whether these can be caused by trazodone only or due to interactions with other drugs.

## Conclusion

In conclusion, we present a case of reversibly induced parkinsonism caused by a moderate dosage of trazodone in a middle-aged male. Nevertheless, interactions with losartan which is metabolized via CYP3A4 and so is trazodone although highly unlikely, couldn't be excluded. Reporting such cases is essential to spread awareness of adverse effects that are thought to be rare or specific for only a few selected groups. Nonetheless, serious adverse trazodone effects such as parkinsonism should be considered in elderly people or those with comorbidities. Drug interactions should always be considered, and regimens adequately adjusted to avoid potentially preventable adverse effects.

## Data Availability

The data used and/or analyzed during the current case report are available from the corresponding author upon reasonable request.

## Abbreviations

CT: Computed tomography
CYP3A4: Cytochrome P3A4
DAT: Dopamine transporter
EEG: Electroencephalography
HBsAg: Hepatitis B surface antigen
HCV: Hepatitis C virus
HIV: Human immunodeficiency virus
MRI: Magnetic resonance imaging
SPECT: Single-photon emission tomography
VDRL: Venereal disease research laboratory test; Syphilis
EPS: Extrapyramidal symptoms
